# Breast Cancer Metastasis with a Ureteral Obstruction and Bladder Mass

**DOI:** 10.7759/cureus.103428

**Published:** 2026-02-11

**Authors:** Sasha Mainer, Hannah Lachmayr, Benjamin Lemon, Wylly Killorin

**Affiliations:** 1 Urology, Mercer University School of Medicine, Columbus, USA; 2 Urology, Mercer University School of Medicine, Valdosta, USA; 3 Urology, St. Francis Hospital, Columbus, USA

**Keywords:** breast cancer metastasis, hydronephrosis, invasive ductal carcinoma, metastasis to bladder, metastatic breast carcinoma, ureteral obstruction

## Abstract

Breast cancer is the most common cause of cancer mortality in females globally, and a significant proportion of patients develop metastatic disease. Common metastatic sites of breast cancer include bone, lung, liver, and brain. Secondary neoplasms (metastases from other primary sites) represent a minority of all malignant bladder tumors

In this report, we describe a case of breast cancer metastasis with ureteral obstruction and bladder mass in a 60-year-old African-American female. For over a decade, this patient was asymptomatic and had been under surveillance following bilateral radical mastectomy for invasive ductal carcinoma (IDC) with adjuvant chemoradiotherapy. Unrelated imaging revealed an incidental finding of right hydronephrosis. Subsequent cystoscopy revealed a bladder mass obstructing the right ureteral orifice. Transurethral resection of a bladder tumor and ureteral stent placement were performed, and the pathology findings favored metastatic breast cancer.

The details of the case are intended to help further knowledge of urinary bladder metastases. This case is unusual in that the patient was asymptomatic, her breast cancer type is associated with a lower incidence of bladder metastases, and the bladder was the solitary location of metastasis.

## Introduction

Breast cancer is the most common cancer in women globally. About one in eight women in the United States will be diagnosed with invasive breast cancer, and the median age at the time of breast cancer diagnosis is 62 years [1]. The five-year relative survival rate of all stages of breast cancer is 91%, while that of distant stages is 32% [1]. Black and American Indian/Alaska Native women are most likely to be diagnosed with regional- or distant-stage breast cancer, rather than localized-stage breast cancer [1].

Most breast cancer is sporadic (90% to 95%), and only 5% to 10% of patients have an identifiable genetic mutation [2]. Gene mutation is most common in the genes BRCA1 and BRCA2 [2]. The histologic types of invasive breast cancer include ductal adenocarcinoma, lobular carcinoma, mucinous carcinoma, tubular carcinoma, and medullary carcinoma. Invasive ductal carcinoma (IDC) comprises 50% to 75% of all invasive breast cancers. Breast cancer subtypes can be further classified based on immunohistochemical expression of hormone receptors, which include estrogen receptor positive (ER+), progesterone receptor positive (PR+), and human epidermal growth factor receptor positive (HER2+). Additionally, the Ki-67 antigen is a cellular marker of proliferation.

Breast cancer metastases are not uncommon. Though only 6% of breast cancers are metastatic at the time of diagnosis [3], about 30% of patients initially diagnosed with earlier stages of breast cancer eventually develop recurrent or metastatic disease. Common breast cancer metastatic sites are the bone, lung, liver, and brain [4-5]. Metastases to the bladder are rare occurrences and account for approximately 2% of all breast cancer metastatic sites [6]. 

This report details ureteral obstruction with a solitary bladder mass seen in a 60-year-old female patient under surveillance for breast cancer. Although urinary bladder metastases from breast cancer typically occur in the setting of widespread metastatic disease, this patient developed an isolated bladder metastasis after approximately 12 years of surveillance. The patient’s bladder mass demonstrated immunohistochemical markers of breast cancer, favoring metastatic disease from IDC. This presentation is uncommon, as most reported cases of breast cancer metastasis to the bladder arise from invasive lobular carcinoma.

The discovery of an asymptomatic, solitary bladder metastasis from IDC after approximately 12 years of surveillance is clinically significant for the following reasons: This presentation exemplifies the well-documented phenomenon of late breast cancer recurrence. The patient’s long disease-free interval (~12 years) is associated with improved prognosis. A solitary metastasis identifies potential oligometastatic disease with favorable prognostic features. Although urinary bladder metastases from breast cancer typically occur in the setting of widespread metastatic disease, this patient developed an isolated bladder metastasis, which is exceedingly rare. This presentation is uncommon because the bladder metastasis arose from IDC. Lastly, in this case, immunohistochemical confirmation was essential.

## Case presentation

A 60-year-old African-American female patient with a history of bilateral IDC presented asymptomatically with acute kidney injury (AKI) and right hydronephrosis discovered incidentally on ultrasound during routine evaluation. The patient had no urinary symptoms, hematuria, or other clinical manifestations at the time of presentation. A urology consultation was obtained for further evaluation.

The patient was initially diagnosed with right-sided IDC in 2006 and underwent unilateral radical mastectomy with adjuvant tamoxifen and doxorubicin chemotherapy. She was subsequently diagnosed with left-sided breast cancer in 2011 and underwent a left radical mastectomy with adjuvant radiotherapy. Immunohistochemistry of the left breast primary tumor revealed estrogen receptor/progesterone receptor-positive (ER/PR+) and human epidermal growth factor receptor 2-negative (HER2/neu-) tumor cells. No endocrine therapy was administered following the second diagnosis. The patient completed treatment in 2013 and was placed on a surveillance protocol, remaining disease-free for approximately 12 years.

**Figure 1:**
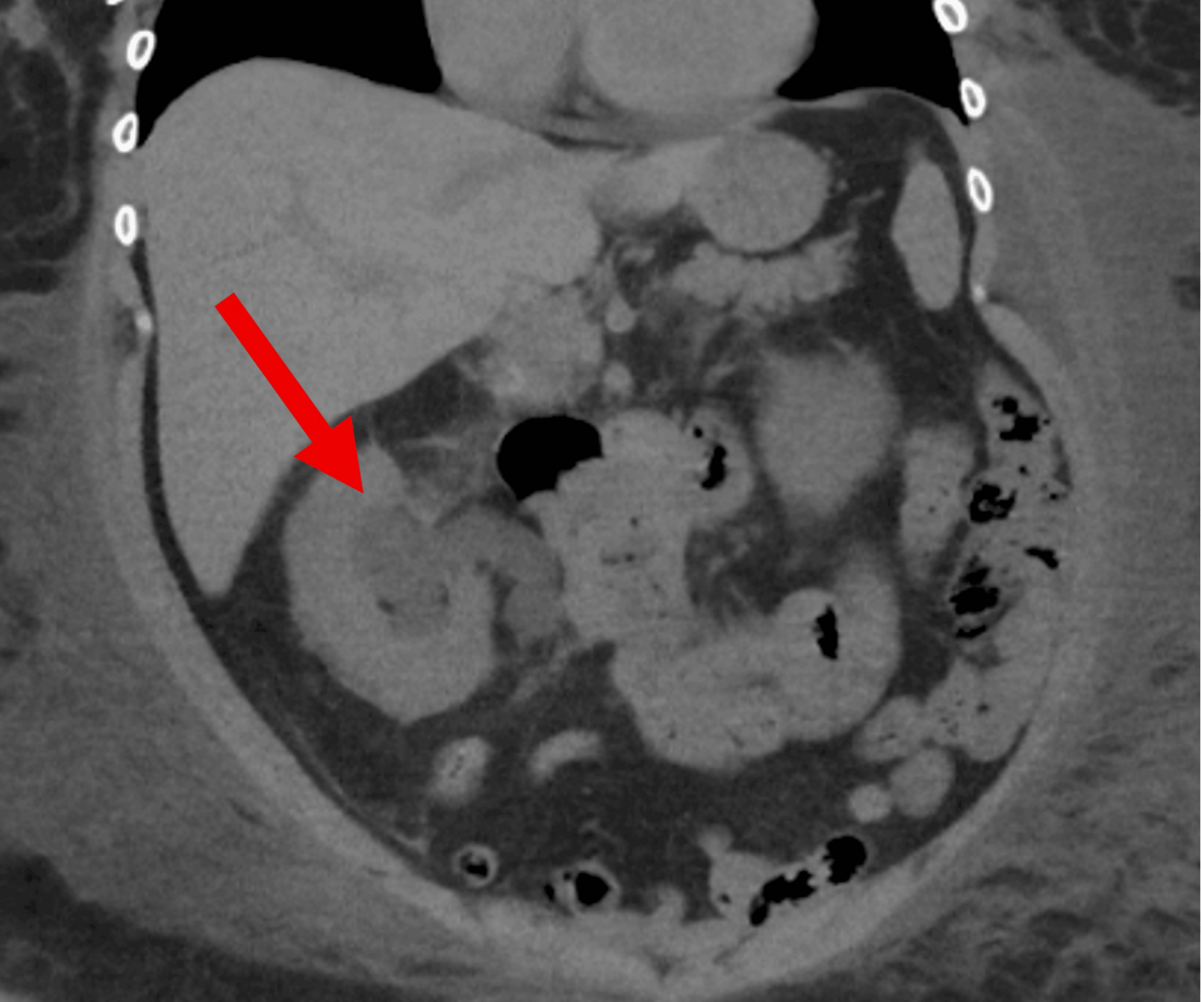
CT of the abdomen and pelvis (coronal) without contrast demonstrating right-sided hydroureteronephrosis (red arrow). CT: computed tomography

**Figure 2:**
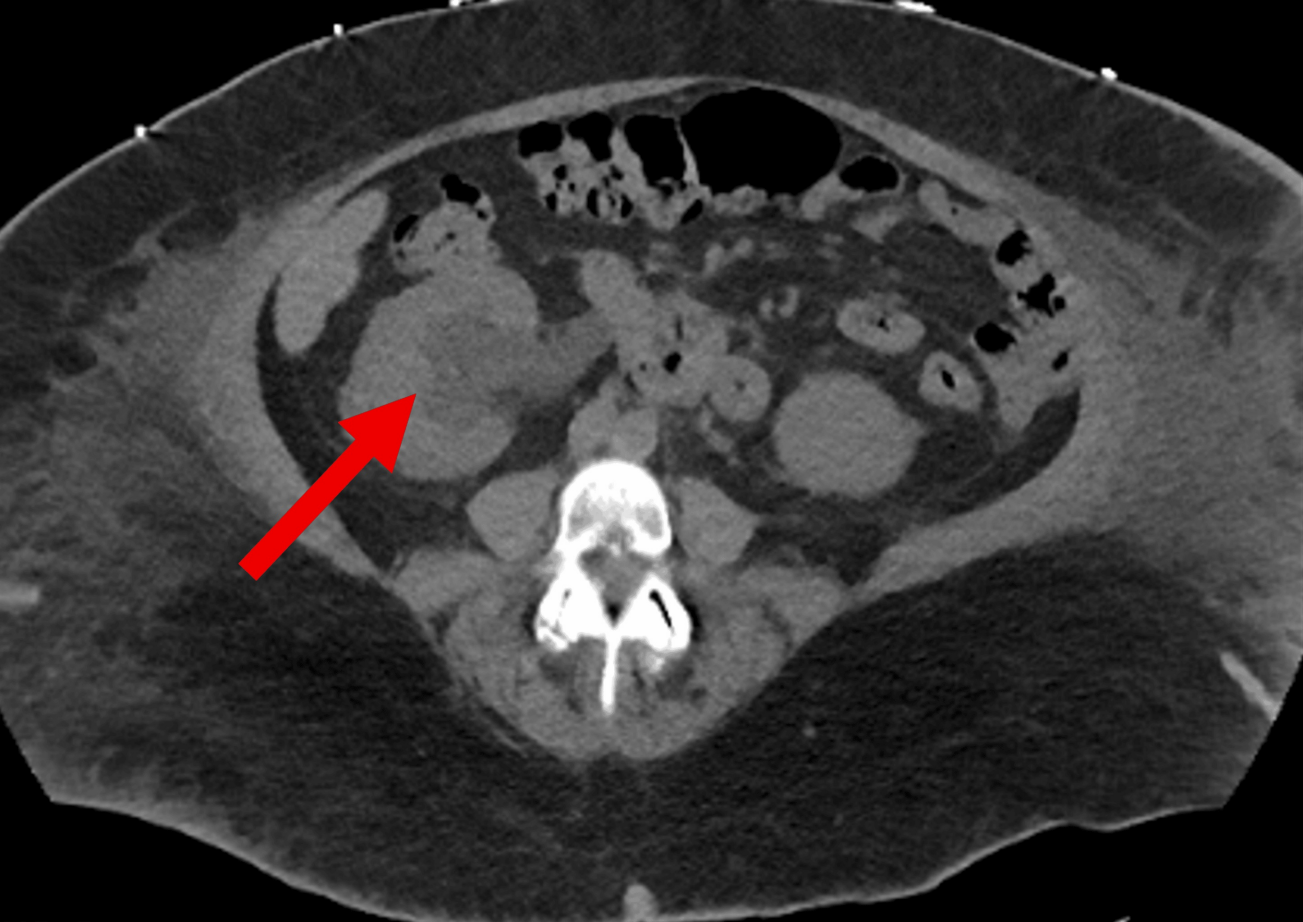
CT of the abdomen and pelvis (axial) without contrast demonstrating right-sided hydroureteronephrosis (red arrow). CT: computed tomography

Computed tomography (CT) of the abdomen and pelvis without contrast revealed moderate right hydroureteronephrosis with a thickened urinary bladder wall (Figures 1-2). Focal wall thickening was noted at the right lateral wall and right aspect of the urinary bladder trigone. The left kidney was unremarkable. Additional findings included diffuse subcutaneous soft tissue edema with a large, localized collection on the left side, minimal pelvic fluid, and left basal pulmonary atelectatic bands.

Given the patient's history of bilateral breast cancer, cystoscopy was performed to evaluate for possible neoplastic bladder involvement. Cystoscopy revealed a large tumor in the right hemi-trigone extending to the right lateral wall with inflamed and irritated overlying mucosa. The right ureteral orifice was not initially visualized due to tumor involvement. Transurethral resection of the bladder tumor (TURBT) was performed. The right hemi-trigone was resected toward the right lateral wall, and the ureteral orifice was subsequently identified. Laser fulguration was used for hemostasis at the tumor base. The resection was performed for diagnostic purposes; complete resection margins were not assessed, and the procedure should be considered a diagnostic biopsy rather than a definitive oncologic resection. A right-sided 6-French, 24 cm ureteral stent was placed for drainage. The procedure was well tolerated without complications. An irrigation catheter was placed, and the Foley catheter was removed on postoperative day four with an uneventful voiding trial.

Pathologic examination revealed tumor cells staining positive for cytokeratin 7 (CK7), ER, HER2/neu, GATA3, and pan-CK. The p63 stain demonstrated differential staining with positivity in the overlying urothelium and glands but complete negativity in the tumor specimen. Cystitis glandularis was also noted. These findings favored the diagnosis of metastatic breast carcinoma rather than primary urothelial malignancy. Notably, the bladder metastasis demonstrated HER2/neu positivity, whereas the patient's primary left breast cancer (2011) was HER2-negative. This represents a clinically significant case of HER2 receptor discordance between the primary tumor and metastatic site.

## Discussion

Breast cancer metastases to the bladder are a rare phenomenon. Roughly 65 cases of breast cancer metastases to the bladder had been reported as of 2020. A review by Karjol et al. found bladder masses were more common in ILC despite IDC being the predominant breast cancer subtype, reflecting ILC’s known propensity for atypical metastatic sites due to loss of E-cadherin [6]. A review done by Malinaric et al. in 2022 found that most patients present with hematuria (39.5%), and the relative five-year survival rate is 2% [7].

Notably, this patient presented asymptomatically, with the bladder mass discovered incidentally during evaluation of AKI and hydronephrosis. This contrasts with the typical presentation described in the literature, where hematuria is the most common symptom [7]. The asymptomatic nature of this case shows the importance of maintaining a high index of suspicion for metastatic disease in patients with a history of breast cancer, even in the absence of classic urinary symptoms. The incidental discovery via imaging for unrelated findings (AKI workup) demonstrates that bladder metastases may remain clinically silent until they cause obstructive complications.

The discovery of a solitary bladder metastasis after approximately 12 years of surveillance carries important prognostic and therapeutic implications. The median time from breast cancer diagnosis to bladder metastasis is 5.6 years (range 0-28 years), making this patient’s 12-year interval consistent with the known pattern of late recurrence [8].

A prolonged disease-free interval is associated with improved prognosis. Patients with a metastasis-free interval greater than 10 years have significantly better five-year survival (35% vs. 23% for <5 years) and decreased risk of breast cancer-specific mortality (hazard ratio 0.77) compared to those with earlier recurrence [9]. Additionally, a solitary metastasis is one of the most favorable prognostic factors in oligometastatic breast cancer, with patients having solitary extracranial metastases demonstrating 5-year overall survival rates of 46% [10-11]. This combination of favorable prognostic features (i.e., long disease-free interval and solitary metastasis) suggests this patient may have a more indolent disease course than typical metastatic breast cancer.

Distinguishing metastatic breast carcinoma from primary urothelial carcinoma is critical, as treatment differs substantially. Both tumor types share expression of GATA3 and CK7, creating a diagnostic challenge [12-13]. The immunohistochemical findings in this case (i.e., positive staining for CK7, ER, HER2/neu, GATA3, and pan-cytokeratin, with p63 negativity in the tumor) favor metastatic breast carcinoma over primary urothelial carcinoma.

The combination of ER positivity and p63 negativity is particularly discriminatory. ER expression is essentially absent in urothelial carcinoma, and p63 is a well-established urothelial marker that is negative in breast carcinoma [14-15]. A recommended panel to exclude metastatic lobular breast carcinoma from plasmacytoid urothelial carcinoma includes mammaglobin, ER, and uroplakin II [14]. In this case, the ER positivity and p63 negativity, combined with the clinical history of bilateral breast cancer, strongly support the diagnosis of metastatic breast carcinoma.

A pivotal finding in this case is the HER2 receptor discordance between the primary tumor (HER2-negative) and the bladder metastasis (HER2-positive). This conversion has profound therapeutic implications. HER2 discordance occurs in approximately 10% to 28% of patients with metastatic or recurrent breast cancer [16-17]. Mechanisms such as tumor evolution under selective pressure, intratumoral heterogeneity, and technical factors may explain this discordance.

Therapeutic implications of this HER2 conversion are substantial. The HER2-positive status of the metastatic lesion renders this patient eligible for HER2-targeted therapies, including trastuzumab-based regimens, pertuzumab, and antibody-drug conjugates such as trastuzumab deruxtecan [18]. The National Comprehensive Cancer Network (NCCN) guidelines recommend biopsy of at least the first recurrence of disease and evaluation of ER/PR and HER2 status, noting that receptor status can change with treatment and metastatic progression [3]. This case highlights the critical importance of re-biopsying metastatic lesions to reassess receptor status, as the treatment paradigm shifts significantly based on HER2 status determination at the metastatic site.

The NCCN guidelines recommend biopsy to guide treatment of metastatic lesions [19]. Though the American Urological Association (AUA) does not have specific guidelines for non-bladder metastases to the bladder, the guidelines for non-muscle invasive bladder cancer (i.e., imaging, cystoscopy, and TURBT) were appropriately applied in this patient’s case [19].

For a patient with ER-positive, HER2-positive metastatic breast cancer, NCCN guidelines recommend systemic therapy with HER2-targeted therapy, with or without endocrine therapy [19]. First-line treatment options include HER2-targeted therapy + chemotherapy; HER2-targeted therapy + endocrine therapy; and endocrine therapy + CDK4/6 inhibitor [18-19]. NCCN guidelines note that current data do not support ablative metastasis-directed therapy for extending overall survival in oligometastatic breast cancer [3].

In this case, TURBT was performed for diagnostic purposes and to relieve ureteral obstruction, with ureteral stent placement for drainage. The procedure should be considered a diagnostic biopsy rather than a definitive oncologic resection, as complete resection margins were not assessed. The decision regarding adjuvant systemic therapy following TURBT should be guided by restaging to confirm oligometastatic status, HER2-positive status of the metastasis, ER-positive status, and multidisciplinary discussion.

This case report depicts the evaluation, diagnosis, and treatment of a rare, solitary bladder metastasis from IDC discovered incidentally after approximately 12 years of surveillance. The asymptomatic presentation, prolonged disease-free interval, and solitary nature of the metastasis represent favorable prognostic features. The critical finding of HER2 receptor discordance, with conversion from HER2-negative in the primary tumor to HER2-positive in the metastasis, fundamentally changes the treatment approach and emphasizes the importance of re-biopsying metastatic lesions. This case reinforces guideline recommendations for mandatory receptor re-evaluation at metastatic sites and highlights the need for multidisciplinary coordination in managing oligometastatic breast cancer.

## Conclusions

This case describes a rare presentation of metastatic breast cancer: an asymptomatic, solitary bladder metastasis from IDC discovered incidentally after approximately 12 years of surveillance. Several features make this case clinically significant. First, bladder metastasis from breast cancer is exceedingly rare, and an isolated bladder metastasis without disseminated disease is even more unusual. Additionally, the metastasis arose from IDC, whereas ILC is disproportionately represented in bladder metastases due to its propensity for atypical metastatic sites. The asymptomatic presentation contrasts with typical findings, as hematuria is the most common presenting symptom. The combination of a prolonged disease-free interval and solitary metastasis represents favorable prognostic features. Critically, the HER2 receptor discordance (i.e., conversion from HER2-negative in the primary tumor to HER2-positive in the bladder metastasis) fundamentally changes the treatment paradigm by rendering the patient eligible for HER2-targeted therapies. This negative-to-positive conversion occurs uncommonly in metastatic cases and reinforces guideline recommendations for mandatory re-biopsy of metastatic lesions to reassess receptor status.

This case highlights the importance of maintaining a high index of suspicion for metastatic disease in patients with a history of breast cancer presenting with unexplained urologic findings, obtaining tissue for pathologic confirmation and receptor re-evaluation, and coordinating multidisciplinary care to optimize treatment based on the metastatic tumor's receptor profile.
